# Perinatal maternal depression and cortisol function in pregnancy and the postpartum period: a systematic literature review

**DOI:** 10.1186/s12884-016-0915-y

**Published:** 2016-05-31

**Authors:** Sunaina Seth, Andrew J. Lewis, Megan Galbally

**Affiliations:** School of Psychology, Deakin University, Melbourne, 3125 Australia; School of Psychology and Exercise Science, Murdoch University, Perth, 6150 Australia; School of Medicine, University of Notre Dame, Perth, Western Australia 6959 Australia; Fiona Stanley Hospital, Perth, 6150 Australia; Harry Perkins South Medical Research Institute, Perth, Western Australia 6009 Australia

**Keywords:** Cortisol, Steroid hormones, Perinatal depression, Postpartum depression, Postnatal depression, Antenatal depression, Maternal blues, Baby blues, Pregnancy, Maternal mood

## Abstract

**Background:**

Perinatal depression has a significant impact on both mother and child. However, the influence of hormonal changes during pregnancy and the postpartum period remains unclear. This article provides a systematic review of studies examining the effects of maternal cortisol function on perinatal depression.

**Method:**

A systematic search was conducted of six electronic databases for published research on the relationship between cortisol and perinatal depression. The databases included; MEDLINE complete, PsychINFO, SCOPUS, Psychology and Behavioural Sciences, Science Direct and EBSCO, for the years 1960 to May 2015. Risk of bias was assessed and data extraction verified by two investigators.

**Results:**

In total, 47 studies met criteria and studies showed considerable variation in terms of methodology including sample size, cortisol assays, cortisol substrates, sampling processes and outcome measures. Those studies identified as higher quality found that the cortisol awakening response is positively associated with momentary mood states but is blunted in cases of major maternal depression. Furthermore, results indicate that hypercortisolemia is linked to transient depressive states while hypocortisolemia is related to chronic postpartum depression.

**Discussion and Conclusion:**

Future research should aim to improve the accuracy of cortisol measurement over time, obtain multiple cortisol samples in a day and utilise diagnostic measures of depression. Future studies should also consider both antenatal and postnatal depression and the differential impact of atypical versus melancholic depression on cortisol levels, as this can help to further clarify the relationship between perinatal depression and maternal cortisol function across pregnancy and the postpartum period.

**Electronic supplementary material:**

The online version of this article (doi:10.1186/s12884-016-0915-y) contains supplementary material, which is available to authorized users.

## Background

The mental health of women during the perinatal period is affected by many factors including genetic predisposition, past history of mental illness, anxious temperament, lack of family or social support and stressful life events [1–4]. Since pregnancy brings major alterations in the levels and function of key endocrine systems, the role of endocrine changes across the perinatal period has been widely investigated as an influence on maternal mood and behaviour [5] as well as fetal and child development [6, 7]. The current paper aims to review the literature examining cortisol functioning in relation to perinatal depression.

Perinatal depression refers to the experience of major or minor depressive episodes during pregnancy or within the first 12 months postpartum. It is highly prevalent, effecting 7–13 % of pregnant women and 10–15 % of women in the six months following childbirth [8]. Furthermore, depression in the perinatal period has been linked to a range of negative outcomes such as social isolation [9], marital discord [10], child delays in motor or intellectual development [11], restricted fetal growth and elevated stress reactivity in infants [12, 13]. It is particularly notable that vulnerability to mental health problems in later life increases for infants born to mothers with postpartum depression [14] and a number of mechanisms have been proposed for this transmission [15]. More specifically, Murray et al. (2011) report findings from a cohort study where 41.5 % of children with depressed mothers experienced depression by age 16 in comparison to 12.5 % of children with non-depressed mothers [14].

### Cortisol and the stress response

Stress plays a key role in the onset and persistence of depression. Cortisol is released in response to stress and is a key physiological marker for activation of the stress response [16]. There has been substantial debate concerning the role of cortisol in depression in general and specifically during pregnancy and the postpartum [16, 17]. Cortisol is a glucocorticoid steroid hormone, synthesized from cholesterol in the adrenal cortex and its release is regulated via the hypothalamic-pituitary-adrenal (HPA) system [18]. Typically, in response to the cognitive appraisal of a significant stressors (which can be real or imagined), corticotrophin releasing hormone (CRH) is produced in the paraventricular nucleus of the hypothalamus and released into the pituitary gland [19]. CRH then stimulates the release of adrenocorticotropic hormone (ACTH) in the anterior pituitary which subsequently results in the adrenal cortex releasing a number of glucocorticoids, including cortisol in humans [19]. The HPA axis operates in a negative feedback loop wherein cortisol release returns to hypothalamic and hippocampal brain regions triggering the cessation of CRH release.

In the early phases of a stressor, cortisol promotes an adaptive response by motivating behaviours which have high survival value such as alertness, vigilance, arousal and attention [20]. Cortisol binds to two distinct receptors whose actions regulate the duration and intensity of the stress response. Mineralocorticoid receptors (MR) in the limbic brain region help mediate cortisol’s response by acting at the membrane level during the initial excitatory phase initiated by a stressor to determine an appropriate threshold for cortisol secretion and subsequently regulate gene transcription [20]. In later phases of the stress response, glucocorticoid receptors (GR) mediate termination of the response, suppress information that is unrelated to the initial stressor, and promote recovery through mobilization of energy sources [20]. Therefore, when cortisol secretion is sufficiently high, CRH output is reduced, which in turn, lowers levels of pituitary ACTH and adrenal cortisol [18].

Such a complex cascade of endocrine functions lends itself to numerous points of dysfunction. For instance, the stress response can cause hypercortisolemia (an excess excretion of CRH and cortisol) [18, 21] and a substantial body of research suggests that this excess secretion of cortisol increases vulnerability to depression [22].

### Cortisol in pregnancy

Given the general links between stress, cortisol and depression, it is interesting to consider how pregnancy itself interacts with maternal cortisol and in turn, increases susceptibility to depressive symptoms in the perinatal period. First, the placenta is a major endocrine organ that produces CRH as it develops, which adds to CRH production in the mother’s hypothalamus. Placental and hypothalamic CRH are broadly similar in relation to their structure, bioactivity and immune-reactivity. However, distinct from the role of cortisol in the negative feedback system of the HPA axis, maternal cortisol release promotes stimulation of CRH in the placenta instead of suppressing it [19]. As a consequence, placental CRH and cortisol in maternal plasma increases exponentially across pregnancy and maternal levels can be 60 to 700 times higher than prior to pregnancy [23]. In response to this high level of cortisol, women with a well-functioning stress response become less responsive to external stressors during pregnancy through reduced activation of CRH neurons in the parvocellular paraventricular nucleus [24]. This lowered activation is due to brainstem afferents being less effective in stimulating CRH neurons (for physical stressors) and altered processing of limbic structures (for emotional stressors) during pregnancy [25]. In women with a dysregulated HPA axis, it is suspected that this attenuation fails to occur and high levels of cortisol secretion common in pregnancy may then lead to hypercortisolemia [21]. In turn, hypercortisolemia can increase a woman’s risk of developing depressive symptoms [22], possibly in the context of environmental stressors.

Alternatively, a second possible mechanism could be that withdrawal from an excess level of cortisol during pregnancy instigates depression in the postpartum due to hypocortisolemia (where the adrenal cortex secretes less cortisol than needed) [26]. The typical pattern of cortisol levels commonly seen in pregnancy involves a gradual increase, peaking at delivery and a sharp decline to baseline level within the first three days postpartum [27]. This drop not only represents the absence of placental cortisol but also reflects a transient suppression of hypothalamic CRH. A number of researchers have suggested that the body normally self-adjusts to this withdrawal within the postpartum period but in cases of postnatal depression, an over-adjustment occurs, which leads to hypocortisolemia and triggers depressive symptoms [28, 29]. It is hypothesised that this relationship between hypocortisolemia and maternal mood is mediated by interactions between cortisol and the dopaminergic systems [30–32].

### Measurement of cortisol

Cortisol in humans is secreted diurnally, with the normal pattern being higher levels at waking, a significant increase in cortisol concentrations 30–45 min after waking and a subsequent decline across the remainder of the day, reaching its nadir at midnight [33]. The increase after waking is referred to as the Cortisol Awakening Response (CAR) and is typically represented by the difference between cortisol levels upon awakening and 30–45 min after waking [34]. CAR can be measured to obtain peak cortisol levels and an accurate representation of the diurnal pattern of cortisol is typically obtained from measurement of multiple cortisol samples coinciding with the normal circadian pattern across the day [33].

Cortisol can be measured through different substrates (blood, saliva, hair, urine) and measurements of cortisol concentrations may vary based on the substrate being used. For example, approximately 80 % of total serum cortisol is bound to cortisol-binding globulin and albumin (plasma proteins), while free cortisol (saliva and urine) is not protein bound [33]. Thus, one possible reason for differences in cortisol measurements between substrates can be attributed to changes in cortisol-binding globulin and albumin altering serum cortisol concentrations, without effecting free cortisol levels [35]. Furthermore, Kirschbaum and Helhammer et al. (1989) [35] suggest that absolute values of cortisol found in saliva are usually lower than blood serum due to an enhanced conversion of cortisol to cortisone (an inactive metabolite) in saliva. In terms of urine samples, Jung et al. (2014) indicate that 24-h urine samples capture average cortisol levels but fail to identify fluctuations in concentrations across the day or the peaks and troughs following dosing [36]. Overall, each substrate has a set of strengths and limitations that can influence measurements of cortisol concentrations and the choice of substrate needs to be consistent with the research question posed.

### Aims of the current review

To our knowledge this is the first systematic review of the literature focusing solely on studies investigating cortisol and perinatal depression. Previous reviews have examined selected studies, with most of these papers considering cortisol in conjunction with other endocrine factors [37–43]. Kendell et al. (1985) and Wieck et al. (1989) reviewed five studies exploring the direct relationship between cortisol and postpartum depression, reporting results as mixed and inconclusive. Smith et al. (1991) reviewed two studies [44, 45] and suggested that there may be a positive association between cortisol levels and perinatal depressive symptoms. Following this, Harris et al. (1996) described three of their own studies [46–48] related to cortisol and perinatal depression, but did not form any conclusions. In contrast, Hendrick et al. (1998) reviewed five [44, 46, 48–50] studies and reported no significant association between cortisol and postpartum depression. They also found notable confounding variables amongst the literature and suggested that further research is required. McCoy et al. (2003) then examined two studies [51, 52] relating to perinatal depression and CRH or cortisol, and found hypocortisolemia to be predictive of postpartum depression. Similarly, Glynn et al. (2013) analysed three studies [2, 53, 54] investigating cortisol in the postpartum period and found lower concentrations in women with postpartum depression, suggesting hypocortisolemia as a related mechanism. Overall, these reviews have either indicated no association or hypocortisolemia in postpartum depression.

In this review we conducted systematic searches of the literature in order to address a number of questions. Firstly, to examine if there is evidence that hypercortisolemia predicts the onset of depression during the perinatal period and secondly, to determine whether there is evidence that hypocortisolemia instigates depressive symptoms in the postpartum, due to withdrawal of the placenta. Lastly, the review aims to critically examine the methodology of existing studies and recommend improvements for future research.

## Method

A systematic search was conducted of 6 electronic databases; MEDLINE complete, PsychINFO, SCOPUS, Science Direct, Psychology and Behavioural Sciences, and EBSCO up until 23/05/2015. The following search terms were used; cortisol, postnatal depression, postpartum depression, antenatal depression, perinatal depression, depressive disorder, depressi*, depression, pregnancy, maternal blues, depressive symptoms (see Additional file 1 for the Scopus search syntax). One investigator selected studies according to the inclusion and exclusion criteria below and this selection was then verified against the source by a second investigator.

### Inclusion and exclusion criteria

The review included studies presenting associations between cortisol levels and depression within the perinatal period or postpartum blues. Perinatal depression is defined as diagnosed major depression with perinatal onset, typically measured on a validated diagnostic instrument during pregnancy or up to one year postpartum. Postpartum blues refers to a transient mood lability characterized by mild depressive symptoms, lasting up to 14 days postpartum. The review accepted measurement of cortisol in any valid substrate and this included saliva, hair, urine or blood-serum. Studies were excluded if they involved non-human participants or focussed on paternal depression, infant hormone levels, genetic research, and conditions other than depression or hormones other than cortisol. Reviews of the literature, or studies that focused on the theoretical underpinnings of cortisol were also not included.

### Selection of studies

**Fig. 1:**
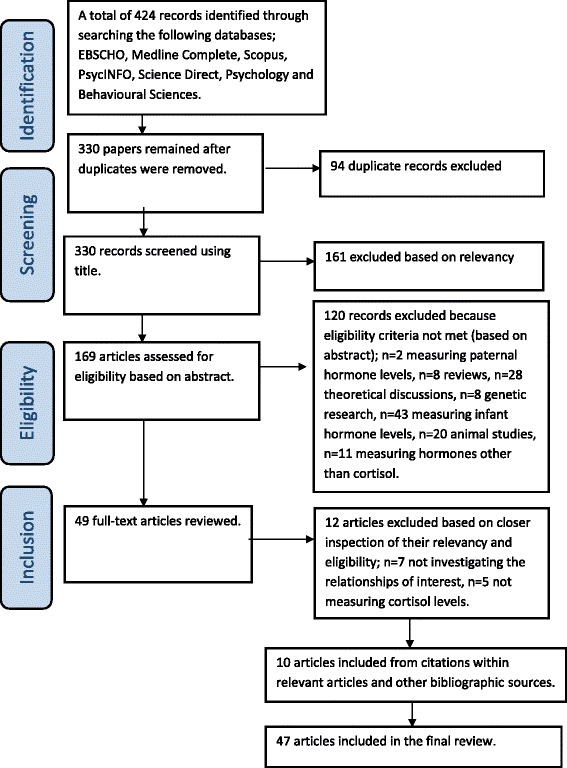
Flowchart of systematic literature review

There were a total of 330 papers identified from all database searches after duplicates were removed. The papers were initially screened through application of the inclusion criteria to titles and then to abstracts. As a result, 281 papers were excluded and 49 papers remained to be considered. In a second level of screening, full papers were reviewed and assessed against the inclusion criteria, with 37 meeting the specified requirements. A further 10 papers were found from citations within these 37 papers and other bibliographic sources, resulting in a total of 47 relevant papers (see Fig. 1).

### Data extraction and manipulation

All three authors examined the selected papers, extracted relevant data and critically examined paper content. Effect sizes were calculated as *R*^2^ when this statistic was not explicitly stated in papers. The following equations were used to calculate *R*^2^:

$$ {R}^2 = {\left[d/\ \left(\surd\ \left({d}^2\right) + 4\right)\right]}^2 $$

where *d* = *M*_1_ − *M*_2_/ √ *SD*_1_^2^ + √ *SD*_2_^2^/2 or alternatively:

$$ {R}^2={t}^2/\ \left({t}^{2 + }df\right) $$

where the t-statistic was provided [55]. In these equations, *d* = Cohen’s d value (standardized mean difference); *M*_1_, *M*_2_ = Mean values of the groups being compared; *SD*_1_, *SD*_2_ = Standard deviation of the groups being compared; *t* = *t*-test statistic, *df* = degrees of freedom. The current review did not report effect sizes for studies with insufficient data to calculate *R*^2^.

## Results

Based on the search criteria outlined above, 47 studies were identified and are summarised in Table 1. Overall 24 studies have reported significant associations between cortisol and depressive symptoms while another 23 did not find significant associations. The first published study was by Handley et al. (1977) and investigated the association between tryptophan, cortisol and mood in 18 healthy postpartum women. Both participant mood and hormone levels from blood plasma were measured once daily 2–5 days postpartum between 9:00–9:30 am and mood was identified using three self-rating scales; The Multiple Affect Adjective Check List (MAACL), Beck Depression Inventory (BDI) and Hildreth Feeling Scale (used to detect elevated mood). The authors found a significant positive relationship between cortisol and elevated mood. In contrast, the same authors conducted a second larger study [44] with 71 participants and found that cortisol levels measured once daily at 9:00–9:30 am, from 38 weeks gestation to 5 days postpartum, were consistently higher in cases of depression than controls. However, this second study reported that seasonal variations in plasma cortisol made it difficult to interpret their findings as there were a significantly greater number of depressed women during the time of year when cortisol was high. Although these two constructs may be causally related, this is difficult to establish [44]. After taking the seasonal factors into account, the study reported an elevation in cortisol only at 38 weeks gestation in women with depressive symptoms.

**Table 1:** Summary of existing literature exploring the association between cortisol and perinatal depression

Authors	Subjects:	Design	Measurement of cortisol (IV)	Depression and stress reactivity measure (DV)	Relevant findings
1. Handley et al. (1977) [70]	*N* = 18 healthy pregnant women	Cohort Study 1 sample at 4 time points: 2, 3, 4, 5 days postpartum. Time: 9–9:30 am	Blood plasma, method of cortisol determination unspecified	MAACL, BDI, Hildreth Feeling Scale, the Blues Index devised by Handley et al. (1980).	Plasma cortisol was significantly and positively correlated with the Hildreth Feeling scale (*R* ^2^ = 0.12, *p* < 0.05).
2. Handley et al. (1980). [44]	*N* = 71 healthy pregnant women	Longitudinal study 1 sample at 9 time points: 36, 38 weeks gestation, 1 to 5 days postpartum, at delivery and 6 weeks postpartum. Time: 9:00–9:30 am	Blood plasma, method of cortisol determination not specified.	MAACL, VAS,BDI Global Ranking Scale Blues Index (Handley et al. 1980)	Cortisol was higher in “cases” and “severe cases” of depression than non-cases from 38 weeks gestation to 5 days post-partum. Global Ranking Scale: *R* ^2^ = 0.16, MAACL: *R* ^2^ = 0.14, VAS: *R* ^2^ = 0.19, BDI: *R* ^2^ = 0.12, Blues Index = N/S.
3.Balbi et al. (1980) [74]	*N* = 25 healthy pregnant women	Cohort Study 4 samples at 1 time point: on 4 days postpartum, a sample was retrieved every 6 hours for 24 hours.	Blood plasma, RIA	HAMD	The depressed group (*n* = 6) had significantly higher cortisol levels than controls.
4. Kuevi et al. (1983). [75]	*N* = 44 healthy pregnant women	Cohort Study 1 sample from 4 time points: 2,3,4,5 days postpartum and for *N* = 35, 2–3 hrs after the last breast feed. *N* = 18 also had an antenatal sample at 36 weeks gestation. Time: 10:00 am–12:00 pm	Blood plasma, RIA	A questionnaire including; self-rating mood scale, VAS, and questions on the frequency and duration of crying.	No significant relationship between mood and cortisol was found.
5.Brinsmead et al. (1985) [76]	*N* = 19 healthy pregnant women.	Cohort Study 1 sample from 3 time points: 36–38^th^weeks gestation, during labor, 4 days postpartum. Time not reported	Blood plasma, RIA	POMS, Caroll Depression Inventory, a set of 5 self-rated visual analogue scales	No significant association was found between maternal blues and cortisol.
6. Feksi et al. (1984). [77]	*N* = 5 postpartum primiparous women who experienced severe blues and 5 matched mothers who did not experience any depressive symptoms.	Case controlled pilot study 20 samples: Samples collected at 6 am, 12 pm, 6 pm and 10 pm daily, from day 1 to day 5 postpartum	Saliva, RIA	Semi-structured interview (Pitt, 1973). VAS, DACL	No significant relationship between cortisol and mood was found.
7. Gard et al. (1986) [49]	*N* = 52 healthy pregnant women	Longitudinal study 1 samples at 2 time points: 36–38 weeks gestation and between 1–5 days postpartum. Time not reported.	Blood plasma, method of cortisol determination not specified.	MAACL, BDI, unidentified retrospective antenatal interview assessing mood.	No significant relationship between cortisol and mood was found.
8. Harris et al. (1989) [48]	*N* = 147 postpartum women	Cohort study 3 samples across 2 days at 6–8 weeks postpartum: between 1:30–3 pm, 10 pm and 8 am the next day.	Saliva and blood plasma, RIA.	EPDS, cut-off score: 12 Raskin 3 Area Depression Rating Scales, MADRAS	No significant relationship between cortisol and mood was found.
9. Ehlert et al. (1990). [71]	*N* = 70 postpartum women, 29 developed postpartum blues.	Longitudinal study 15 samples: From the first day after delivery to 5 days postpartum, all women provided 3 saliva samples daily (8 am, 3 pm, 8 pm)	Saliva, RIA	BDI DACL BFS (adjective checklist)	Women who experienced postpartum blues showed significantly higher cortisol levels in the morning on days where symptoms were present, in comparison to women who did not experience the blues (*t*(59) = -2.35, *p* < 0.022, *R* ^2^ = 0.10)
10. Smith et al. (1990) [45]	*N* = 97 primiparous Australian women (28 weeks gestation) Divided into women whose mood either improved (*n* = 46)or deteriorated (*n* = 36) from 38 weeks gestation to 2 days postpartum.	Longitudinal study 1 sample from 4 time points: 28 and 38 weeks’ gestation, during labour and day 2 postpartum. Time: between 8–10 am.	Blood plasma, RIA	POMS MADRS	There were no significant differences in cortisol levels between groups (*F* = 1.75, *p* > 0.05).
11. O’Hara et al. (1991) [46]	*N* = 182 healthy pregnant women.	Longitudinal study 9 time points: At 34, 36, 38 weeks; 3× blood samples daily and 24 hour urine samples collected. 1× blood sample daily on 1, 2,3,4,6, and 8 days postpartum and 24 hour urine samples on 2& 4 days postpartum. Time: “before breakfast” On day 4 postpartum, an additional blood sample was drawn at 4 pm.	Blood plasma and urine, RIA	BDI, VAS, Maternal Blues Index (Handley et al., 1980).	No significant association was found between cortisol and mood.
12. Okano et al. (1992). [52]	*N* = 47 healthy pregnant women Attrition rate: 19 %	Longitudinal study 1 sample from 3 time points: 30–41 weeks, 3^rd^/4^th^ day postpartum and 1 month postpartum. Time: 10 am during pregnancy and 1 month postpartum, 7 am on 3^rd^/4^th^ day postpartum.	Blood plasma, RIA	Semi-structured interview adapted from SADS SRDS, Stein Scale for Maternity Blues	Cortisol levels were significantly higher 3–4 days postpartum in the “blues” group in comparison to those without depressive symptoms, *R* ^2^ = 0.18.
13. Pedersen et al. (1993). [50]	*N* = 12 healthy pregnant women	Case controlled study I sample from 6 time-points: 38 weeks, and 1, 3, 6, 9 and 12 days postpartum. Time not reported.	Urine and blood plasma samples, method of cortisol determination not specified.	VMAS, CSI HRSD	Morning serum cortisol levels were significantly higher 6 days postpartum in the group with depressive symptoms (via HRSD) than controls (*R* ^2^ = 0.08). No significant differences in urinary cortisol between groups at any time point.
14. Taylor et al. (1994) [73]	*N* = 163 healthy postpartum women.	Cohort Study 1 sample, 1 time point: 3 days postpartum. Time: 10:30 am–12:00 pm	Blood plasma, RIA	The Kennerley Blues Scale EPDS, cut-off:10	Cortisol levels were significantly higher in the blues group than non-blues group (as identified by the Kennerley Scale), *R* ^2^ = 0.07.
15. Harris et al. (1994). [78]	*N* = 130 primiparous, healthy pregnant women	Longitudinal study Saliva: 8 am, 10 pm (2 weeks pre-term until 35–40 days postpartum daily) and additional 2 pm samples on 1, 2,3,4,5 days postpartum. Blood samples: 1 sample at 2 weeks before delivery, 1, 5, and 35 days postpartum. Time not reported.	Saliva, RIA	EPDS (did not specify cut-off score), Stein Scale for Maternity Blues, BDI, MADRAS.	There were no significant associations between blues and cortisol (neither mean concentrations at the times of plasma sampling nor the decrements in concentrations from before delivery to day 5 postpartum)
16. Mahomed et al. (1995) [90]	*N* = 189 healthy pregnant, primiparous women	Prospective study 1 sample, 1 time point (cortisol): when in established labour. Time not reported.	Blood plasma, RIA	Pitts Depression Inventory.	No significant associations between mood and cortisol.
17.Harris et al. (1996) [47]	*N* = 130 healthy pregnant, primiparous women	Longitudinal study Saliva: 8 am, 10 pm (2 weeks pre-term until 35–40 days postpartum daily) and additional 2 pm samples on 1, 2,3,4,5 days postpartum. Blood samples: 1 sample at 2 weeks pre-term, 1, 5, and 35 days postpartum. Time not reported	Saliva and Blood, RIA	EPDS (did not specify cut-off score), MADRAS, Stein Scale for Maternity Blue, Raskin 3 Area Depression Rating Scales, a semi-structured interview for depression using DSM-III-R criteria for major depression.	Depressed women had significantly (*p* < 0.05) lower evening (10 pm) cortisol on pre-natal day 14 (using all measures), pre-natal day 1 (using the Raskin, MADRAS and semi-structured interview), pre-natal days 2–7 pooled and 3 days postpartum (using the Raskin & MADRAS), *R* ^2^ = 0.10–0.15.
18. Abou-Saleh et al. (1998). [79]	*N* = 61 women (23 pregnant women and 38 non-gravid controls). 3 groups: postpartum women, pregnant women and controls.	Cross-sectional study 1 sample, 1 time point: Cortisol measured 7 days postpartum, between 9–10 am	Serum cortisol, RIA	EPDS, cut-off score: 11 PSE	There was no significant relationship between cortisol and mood.
19.Lundy et al. (1999) [56]	*N* = 63 pregnant women (36 with depression) Cortisol samples retrieved from a subsample of 43 (25 depressed, 18 non-depressed).	Case controlled study 1 sample at 2 time points: between 27–35 weeks gestation and shortly after term. Time: “morning hours”.	Urine (not 24 hr samples), method of cortisol determination not specified	CES-D, DIS	Depressed mothers had significantly higher prenatal cortisol levels than non-depressed mothers, *F* (1, 42) = 4.16, *p* < 0.05, *R* ^2^ = 0.03.
20. Susman et al. (1999). [80]	*N* = 59 pregnant healthy adolescents (13–19 year olds)	Longitudinal design 1 sample at 3 time points: early pregnancy (8–16 and 9–12 weeks), late pregnancy (32–34 weeks) and 3–4 weeks postpartum. Time: 8:30 am.	Blood plasma, RIA	DISC-2.1(administered across all stages)	No significant relationship was found between cortisol and mood at any time-point.
21. Parry et al. (2003) [53]	*N* = 40, 20 depressed and 20 non-depressed postpartum women	Case controlled study Every 30 minutes from 6 pm to 11 pm, sometime within the first 12 months postpartum.	Blood plasma, Unspecified	HRDS, BD1, EPDS (did not report cut-off score), SCID, VAS.	Hypocortisolemia was indicated in postpartum depressed women, in comparison to controls. Insufficient data to obtain effect size.
22. Field et al. (2004) [57]	*N* = 140 pregnant women (70 depressed, 70 non-depressed)	1 sample at 2 time points: Average. 20.1 weeks gestation, within 24 h following delivery. Time: “morning”	Urine samples, Unspecified	CES-D	Mothers with depressive symptoms had elevated cortisol levels in comparison to controls at 20.1 weeks (on average). *R* ^2^ = 0.05.
23. Diego et al. (2004). [94]	*N* = 80 pregnant women, 23–27 weeks gestation	1 sample at 2 time points: 23–27 weeks gestation, within 2 weeks postpartum. Time: 11 am–1 pm	Urine sample (not 24 hour), RIA	CES-D	Women expressing depressive symptoms during both pregnancy and postpartum and only during pregnancy had significantly higher cortisol levels than non-depressed women during mid gestation (*R* ^2^ = 0.19, *R* ^2^ = 0.31 respectively)
24. Field et al. (2006) [88]	*N* = 300 depressed pregnant women at approx. 20 weeks gestation	Cross-sectional study 1 sample, 1 time point: 20 weeks gestation, Time: “first morning urine sample”	Urine samples, RIA	CES-D, SCID	Cortisol significantly and positively associated with CES-D scores at 20 weeks gestation (*F* = 6.72, *p* = 0.01*, R* ^2^ = 0.02).
25. Nierop et al. (2006). [65]	*N* = 57 healthy multiparous pregnant women	Cross-sectional study 6 samples on a single day: Cortisol samples were measured immediately before and after the TSST and 10, 20, 45 and 60 minutes after testing. Time not reported.	Saliva samples, EIA	Trier Social Stress Test (TSST) EPDS, cut-off score: 9	The group likely to develop depression had greater psychological reactivity to psychosocial stress and greater increases in cortisol levels. Cortisol over time × group effect: *F* (2.41, 25.74) =2.99, *p* = 0.04, *R* ^2^ = 0.05.
26. Groer et al. (2007) [54]	*N* = 25 depressed and 175 non-depressed mothers (at 4–6 weeks postpartum)	Case controlled study 1 sample at 1 time point: Between 4–6 weeks postpartum. Time: before 8 am for saliva and between 8–11 am for blood samples.	Saliva and blood plasma, EIA	POMS-D	Depressed mothers had significantly *lower* salivary cortisol levels than the control group (*p* < .05). Serum cortisol concentrations were not significantly different between groups. Insufficient data to obtain effect size.
27. Davis et al. (2007) [81]	*N* = 247 healthy pregnant women	1 sample at 4 time points: 19.1, 24.9, 30.8 weeks gestation, 8 weeks postpartum. Time: Mean 2:20 pm, SD: 1.5 hrs	Saliva, RIA	CES-D	No significant relationship between mood and cortisol was found.
28. Evans et al. (2008). [66]	*N* = 180 pregnant women at 36 weeks gestation. Based on psychiatric diagnosis, 4 groups were formed: *n* = 121 controls, 16 depressed, 34 had anxiety, and 9 comorbid.	Case Controlled study 1 sample at 3 time points (between 33–39 weeks); upon presentation of task (baseline), before the psychophysiology session started (anticipation) and after the session (reaction). Time: 10:30–11:30 am.	Blood serum, RIA	SCID, CES-D, PES Psychophysiology task: Stroop task, mental arithmetic task or controlled breathing task.	Women with co-morbid depression and anxiety had higher salivary cortisol levels than controls (*p* = 0.01). However those with either depression or anxiety alone did not differ significantly from controls.
29. Field et al. (2008) [59]	*N* = 430 healthy pregnant women	1 sample at 3 time points: approx. 22 and 32 weeks gestation, 2 days postpartum. Time: “mid-morning”	Urine sample, RIA	SCID, CES-D	At 22 weeks gestation, depressed women (as identified by the SCID) had higher cortisol levels than non-depressed women. Insufficient data provided to calculate effect size.
30. Fan et al. (2009). [87]	*N* = 308 pregnant or recently delivered women. *n* = 77 each in 4 groups, representing each trimester and 1 week postpartum.	Cross-sectional study 1 sample at 3 time points: each group (trimester), between 9–10 am	Blood/serum samples, RIA	HAMD, SCL-90	No significant relationship between cortisol and mood was found.
31. Figueiredo et al. (2009). [82]	*N* = 91 healthy pregnant, primiparous women	Longitudinal 1 sample at 2 time points: Between 21–28 weeks and 3 months postpartum. Time not reported	24-hour urine samples, EIA	EPDS, cut-off score: 10	Cortisol was not a significant predictor of maternal depression
32. Yim et al. (2009). [93]	*N* = 100 healthy pregnant women.	Longitudinal study 1 sample at 5 time points: Blood samples were obtained at 15.3, 19.2, 25.0, 31.0 and 36.7 week’s gestation. Time not reported.	Blood plasma, RIA	CES-D, EPDS, cut-off score: 10	At no time during pregnancy were cortisol levels associated with concurrent depressive symptoms or postnatal depression (*p* > .53 for all comparisons)
33. Diego et al. (2009) [63]	*N* = 80 pregnant women (40 depressed, 40 non-depressed)	Longitudinal study 1 sample at 1 time point: between 18–20 weeks gestation. Time: “mid-morning”	Urine, RIA	SCID, CES-D	Depressed women had significantly higher prenatal cortisol concentrations than non-depressed women (determined by the SCID & CES-D), *F* (1, 74) =7.92, *p* = 0.006, *R* ^2^ = 0.14.
34. Cheng et al. (2010) [67]	*N* = 46 healthy pregnant women at or over 36 gestational weeks.	Longitudinal 2 samples at 2 time points: 36 weeks gestation and 4–6 weeks postpartum. Cortisol was collected at waking & 30 minutes after awakening.	Saliva, method of cortisol determination unidentified	CES-D	No significant relationship between prenatal or postnatal CAR and CES-D scores.
35. Taylor et al. (2009) [2]	*N* = 21 depressed and 30 non-depressed women at 7.5 weeks gestation	Cohort study Samples obtained 30 min, 3 and 12 hours post-waking for 2 consecutive days at 7.5 weeks postpartum.	Saliva, EIA	EPDS, cut-off score: 13	Depressed women had a significantly reduced morning rise (at 30 minutes post-waking) in cortisol concentrations than controls, *R* ^2^ = 0.34.
36. Pluess et al. (2010). [68]	*N* = 66 healthy pregnant women	Longitudinal study 4 time points: 35 and 36^th^ gestation weeks, 2 consecutive days during 10–12 weeks gestation. Samples obtained immediately, 30, 45 and 60 minutes after waking.	Saliva, EIA	EPDS, cut-off score: 13	No significant relationship between cortisol and CAR was found.
37. Parcells, D.A. (2010) [60]	*N* = 59 healthy pregnant women	Longitudinal Study 1 sample at 2 times points (cortisol): 26–28 and 32–34 weeks gestation. Time: between 10:00–11:30 am.	Saliva, STAT Fax 2100 microplate reader	SCID,BDI-II	No significant association between SCID diagnoses and cortisol. However, cortisol significantly differed between women with BDI-II scores greater than 12 and less than 12. Insufficient data to obtain effect size.
38.O’Keane et al. (2011). [83]	*N* = 70 healthy pregnant women	Longitudinal study 1 sample at 2 time points: 36 weeks and 3 days postpartum. Time: 11.00 am and 3 pm.	Blood plasma, EIA	EPDS, cut-off score:11 28 item Blues Questionnaire (Kennerley & Gath, 1989)	No significant association between cortisol and depression (antenatal or postnatal) was found.
39. Giesbrecht et al. (2012) [61]	*N* = 83 healthy pregnant women	Longitudinal Study 3 consecutive days between 6–37 weeks gestation with the following sampling schedule; upon waking, 30–45 min after waking and semi-randomly with the anchor times of 11:00 am, 4:00 pm, and 8:00 pm	Saliva, EIA	POMS-15, EDPS, did not specify cut-off score.	CAR and negative mood were significantly associated (after accounting for the diurnal variations across the 3 days), *R* ^2^ = 0.29.
40. Tsubouchi et al. (2011) [84]	*N* = 69 healthy pregnant women	1 sample at 5 time points: 1^st^trimester (10–12 weeks), 2^nd^trimester(20–22 weeks), early 3^rd^trimester(30–32 weeks), late 3^rd^trimester(37–39 weeks) and 1 month postpartum. Time: between 9:00 am and 1 pm.	Saliva, EIA.	Zung self-rating depression scale (cut off score: 42) General Health Questionnaire -28	Participants identified as “chronically stressed” had lower cortisol levels during the 2^nd^ and 3^rd^ trimesters than controls. However no significant difference was found in the 1^st^ trimester or postpartum. Insufficient data to obtain effect size.
41. Salacz et al. (2012) [89]	*N* = 79 pregnant women in their 36–38th gestational week	Cross-sectional study 1 sample at 1 time point: 36–38th gestational week, before 8 am.	Blood plasma, RIA	BDI-IA	No significant relationship between cortisol levels and mood found
42. Voegtline et al. (2013) [65]	*N* = 112 pregnant women between 24 and 38 weeks gestation.	Longitudinal study 1 sample at 5 time points: 24–26 weeks, 27–29 weeks, 30–32 weeks, 33–35 weeks and 36–38 weeks. Time: between 1–3 pm.	Saliva, EIA	CES-D	Women who reported more depressive symptoms between 30–32 weeks had higher cortisol levels than controls, *R* ^2^ = 0.05, *p* < 0.05).
43. Peer et al. (2013) [69]	*N* = 78 healthy pregnant Canadian immigrant women	4 times per day for 2 consecutive days at 19 weeks gestation: immediately post-waking, 30 and 60 minutes post-waking (CAR). Time: between 9:00 pm–10:00 pm.	Saliva, EIA	EPDS, cut-off score: 12.	Evening cortisol levels were significantly higher in women with high levels of depressive symptoms (*n* = 8) than those with low levels of depressive symptoms (*n* = 45). There were no significant differences for CAR., *R* ^2^ = 0.17.
44. Shelton et al. (2014)	*N* = 105 healthy pregnant women	Cohort Study 1 time point: between 16 and 26 weeks gestation. Time: “before noon” (mean time = 11:25 am).	Blood plasma, EIA	POMS-D	There was no significant relationship between POMS-D scores and cortisol, *R* ^2^ = 0.02.
45. O’Connor et al. (2014) [62]	*N* = 101 healthy pregnant women	Longitudinal Study CAR was measured using five samples collected at; upon waking, 45 min, 2.5 hrs, 8 hrs and 12 hrs post-waking. Two CAR measurements: on average, at 21.26 and 34.15 weeks gestation.	Saliva, EIA	EPD (did not specify cut-off scores), SCID	SCID diagnosis of depression were significantly and negatively associated with cortisol upon initial waking. Insufficient data to obtain effect size.
46. Luiza et al. (2015) [91]	*N* = 50 healthy pregnant women recruited at approx. 11 weeks gestation	Case-Controlled Study 1 sample at 1 time point: urine and blood samples collected between 6–16 weeks gestation. Time: not reported for blood samples & urine samples were obtained “first thing in the morning”.	Urine and Blood plasma, EIA.	EDS, cut-off score: 11	There was no significant relationship between cortisol and EDS scores.
47. Shimizu et al. (2015) [104]	*N* = 65 healthy Japanese postpartum women.	Cohort study 1 sample at 2 time points: 1 month and 4 months postpartum. Time not reported.	Urine samples, Unspecified.	EPDS (Japanese version), cut-off score: 8-9	There was no significant relationship between cortisol and EPDS scores.

This table lists and provides details of existing literature examining the association between cortisol and perinatal depression

*Abbreviations: BDI-IA* Becks Depression Inventory revised, *CSI* Childcare Stress Inventory, *CES-D* Centre for Epidemiological Studies Depression Scale, *DACL* Depressive Adjective Check List, *DIS* Diagnostic Interview Schedule, *DISC- 2.1* Diagnostic Interview Schedule for Children, *EIA* enzyme immunoassay, *EPDS* Edinburgh Postnatal Depression Scale, *EDS* Edinburgh Depression Scale, *HAMD* Hamilton Rating Scale for Depression, *HRSD* Hamilton Rating Scale for Depression, *LES* Life Experiences Survey, *MAACL* Multiple Affect Adjective Checklist, *MADRAS* Montgomery-Asberg Depression Rating Scale, *POMS* Profile of Mood States, *PES* Pregnancy Experiences Scale, *PSE* Present State Examination, *RIA* radioimmunoassay, *SCID* Structured Clinical Inventory for DSM Disorders, *SCL-90* Symptom Checklist-90, *SADS* Schedule for Affective Disorders and Schizophrenia, *SRDS* Zung Self-rating Depression Scale, *VAS* Visual Analogue Scale for Mood and Anxiety, *VMAS* Visual Analogue Mood Scales, *CAR* Cortisol Awakening Response, *TSST* Trier Social Stress Test

### Antenatal depression and cortisol

Out of the 24 studies that reported significant associations, 10 [56–64] identified higher cortisol levels in women with antenatal depression than controls. Specifically, Lundy et al. (1999) noted higher levels in women with depressive symptoms between 27–35 weeks gestation and Field et al. (2004; 2006; 2008) reported a positive association between cortisol and depressive symptoms between 20–22 weeks gestation. Likewise, Diego et al. (2009) found significantly higher cortisol concentrations at 18–20 weeks gestation in depressed women (identified by a structured interview) and a positive relationship (*p* < 0.01) between self-reported depressive symptoms and cortisol levels. Parcells et al. (2010) also reported a positive relationship between self-reported depressive symptoms and cortisol at 26–28 and 32–34 weeks gestation, but failed to find a significant relationship between cortisol and diagnosed major depression. Furthermore, Voegtline et al. (2013) found that although all participants had rising cortisol levels across 24 to 38 weeks of pregnancy, those experiencing depressive symptoms displayed higher concentrations between 30–32 gestational weeks. In contrast, Harris et al. (1996) and Tsubochi et al. (2011) reported lower cortisol concentrations in women with depressive symptoms during the second and third trimesters of pregnancy.

#### Cortisol and stress reactivity

Out of the 24 studies reporting significant findings, two studies [65, 66] used a stress challenge to examine cortisol as an index of stress reactivity in pregnancy. Nierop et al. (2006) administered the Trier Social Stress Test (TSST) during 13–31 weeks gestation and saliva samples were taken 10 min and immediately before and after the task, with an additional five samples taken at 10, 20, 30, 45, and 60 min after the TSST. The researchers found that women with a high risk of developing postpartum depression displayed greater reactivity to stressors and higher cortisol levels. Similarly, Evans et al. (2008) ascertained stress reactivity by administering the Stroop task (a cognitive assessment of executive functioning that induces stress), a mental arithmetic task or a controlled breathing task to pregnant women with either no symptoms, depression, anxiety or comorbid depression and anxiety. They obtained three morning saliva samples after subjects arrived (baseline), just before the task and directly after the task. Evans et al. (2008) found that women with comorbid depression and anxiety exhibited a greater stress response than the other groups but there were no significant differences between controls and the group with depression alone. The differences in results between the two studies can be due to a range of factors, including the use of different stress response tasks and the gestational time targeted. Furthermore, Nierop et al. (2006) measured stress reactivity in women susceptible to depression whereas Evans et al. (2008) based their investigation on psychiatric diagnoses of major depression.

#### Cortisol Awakening Response (CAR)

Furthermore, six studies [2, 61, 62, 67–69] investigated the association between the cortisol awakening response (CAR) and perinatal depression. Out of these studies, O’Connor et al. (2014) found significantly lower cortisol levels at waking, a less sharp decline over the day and higher average cortisol levels overall in pregnant women with major depression. They also reported a similar but weaker (non-significant) relationship between self-report Edinburgh Postnatal Depression Scale (EPDS) scores and cortisol levels. Likewise, Taylor et al. (2009) found lower cortisol concentrations in depressed women 30 min post-waking at 7.5 weeks postpartum. In contrast, Giesbrecht et al. (2012) identified a significant positive relationship between CAR and momentary mood states during 6 to 37 weeks gestation. Furthermore, three studies [67–69] failed to find any significant relationships between CAR and antenatal depression, with Cheng et al. (2010) reporting neither prenatal (36 weeks gestation) nor 4–6 weeks postpartum CAR as significantly related to perinatal depression.

### Postpartum depression and cortisol

Six studies [70–74] have reported a significant positive relationship between postpartum depression or depressive symptoms and cortisol. Specifically, higher cortisol concentrations have been associated with depressive symptoms at four [74], one to five [71], three [73] and six days postpartum. Similarly, Okano et al. (1992) also reported significantly higher cortisol concentrations in women experiencing the blues 3–4 days postpartum. They found that cortisol levels peaked for women with and without the blues. However, on 3–4 days postpartum, serum levels of cortisol in controls began to decrease while cortisol in the postpartum blues group continued to increase. Overall, these results suggest that cortisol is higher in women experiencing depressive symptoms 1–6 days postpartum.

In contrast, numerous studies [45, 59, 67, 75–84] have indicated insignificant associations between cortisol and postpartum depression, while Harris et al. (1996) reported lower cortisol concentrations in women with depressive symptoms during the immediate postpartum period. Likewise, recent studies such as Parry et al. (2003) and Groer et al. (2007) have identified a significant negative relationship between cortisol and depressive symptoms within 12 months postpartum and 4–6 weeks postpartum respectively.

### Cortisol and its association with both antenatal and postpartum depression

Harris et al. (1996) and Diego et al. (2004) were the only studies that identified significant associations across the antenatal and postpartum period. Harris et al. (1996) recruited 130 primiparous women and found a negative relationship between depression (as identified by a semi-structured diagnostic interview) and evening cortisol from 2 weeks pre-term to 10 days postpartum, with effect sizes ranging from *R*^2^ = 0.10–0.15. Similarly, Diego et al. (2004) recruited 60 women who completed the Centre for Epidemiological Studies Depression Scale (CES-D), with an equal number of participants (*n* = 20) in each of the following groups: 1) Controls 2) CES-D ≥ 16 during 23–27 weeks gestation 3) CES-D ≥ 16 at 2 weeks postpartum 4) CES-D ≥ 16 during both the pre and postpartum period. The results revealed that cortisol concentrations were significantly higher in women experiencing depressive symptoms between 23–27 weeks gestation and women with depressive symptoms during both the antenatal and postpartum period, in comparison to non-depressed women. However, the study reported a negligible effect size for the latter finding (*R*^2^ = 0.02), with the former association producing a moderate effect (*R*^2^ = 0.31), suggesting that this relationship is stronger during the antenatal period. Nevertheless, these studies have reported directly opposing findings, with Harris et al. (1996) suggesting a negative relationship between cortisol and depression across the perinatal period and Diego et al. (2004) indicating a positive relationship.

### Critical review of methodological variation across studies

There are significant differences in findings across the identified studies including the direction and effect of the relationship between cortisol and perinatal depression. One potential reason for this inconsistency may be low statistical power due to many studies including only a small number of participants with high depressive symptoms or diagnoses. For example, studies have based their findings on 6 out of 25 [74], 7 out of 120, 5 out of 61 [79], 13 of 65 [69] and 16 out of 132 women experiencing depression or depressive symptoms [66]. Furthermore, five studies [70, 76, 77] have small overall sample sizes of between 10 to 25 subjects.

### Quality of cortisol measurements

Heterogeneous findings may also be due to the quality of cortisol measurements. Research assessing salivary cortisol in epidemiological studies [85] indicates that a minimum protocol for sampling cortisol should obtain three cortisol samples per participant across a single day, a medium standard protocol requires six samples daily or three samples per day over three days and a high standard protocol involves multiple samples per day across several days. These protocols are designed to capture the curvilinear nature of cortisol and represent a range of different standards in cortisol measurement quality [85]. Furthermore, ideally, cortisol levels and gradients across the whole peripartum period should be obtained. However, only some studies have focused on gradients or trajectories in relation to cortisol [44, 45, 72, 80, 83, 86, 87] and most [44–46, 48–50, 54, 56, 57, 60, 63, 66, 70, 72, 73, 75, 76, 78–80, 82–84, 87–95] have not met the minimum protocol, with many basing their findings on a single sample [54, 58, 63, 73, 79, 89–91, 95]. In addition, participants may not adhere to sampling protocols for urine and saliva substrates, which can reduce the reliability of measurements.

Furthermore, the time samples are obtained can also influence quality of cortisol samples. Out of the studies showing significant findings, Voegtline et al. (2013) obtained a single sample between 1:00 pm–3:00 pm and reported an effect size of *R*^2^ = 0.05, and Pedersen et al. (1993) obtained one sample on multiple days, did not identify time of sample collection and reported an effect size of *R*^2^ = 0.08. Given the diurnal characteristics of cortisol, it is possible that single samples retrieved in the afternoon produce underestimated cortisol concentrations and in turn, small effect sizes. In support of this premise, it is notable that in the current review most studies reporting larger effect sizes retrieved cortisol levels in the morning; Handley et al. (1977), Handley et al. (1980) gathered samples between 8:00 am–9:30 am and reported effect sizes of *R*^2^ = 0.12 and 0.16, respectively. Similarly, Okano et al. (1992) found an effect of *R*^***2***^ = 0.18 and obtained cortisol levels at 7:00 am while Diego et al. (2004; 2009) measured samples between 11:00 am and 1:00 pm, and revealed effect sizes of *R*^2^ = 0.31 and *R*^2^ = 0.14, respectively. Thus, studies retrieving samples earlier in the day may produce larger effects due to cortisol concentrations being naturally higher during morning hours. Furthermore many studies [47, 49, 50, 58, 65, 76, 78, 90, 91, 93] have not identified the time of sample collection and given cortisol’s diurnal variations, timing can create significant differences between study findings.

In addition, studies have used different substrates, which can cause variations in measurements of cortisol concentrations. For example, Groer et al.(2007) [54] found that saliva and serum cortisol concentrations were not correlated, with salivary cortisol being significantly lower than serum in depressed women. Furthermore, different biochemistry assays have been used to analyse hormones across the studies reviewed. Some recent studies [2, 54, 61, 62, 65, 68, 69, 82–84, 91, 95] have utilised enzyme-linked immunosorbent assay (ELISA) kits whereas the majority [45, 46, 48, 52, 56, 59, 63, 66, 71, 73–77, 79–81, 86–90, 93, 94] have adopted radioimmunoassay (RIA) techniques. Raff, Homar and Burns (2002) compared the processing of salivary cortisol using ELISA kits and RIA, and found that RIA gave results much closer to the expected value of an independently created cortisol stock solution diluted in saliva. They suggested that salivary cortisol concentrations are substantially higher using ELISA kits, with ELISA’s over-estimating cortisol levels [96]. In contrast, Murphy (2002) found that commercially available RIA’s yielded 2–3 times greater urinary free cortisol than true values obtained from chromatography [97]. Thus, cortisol levels may also reflect the choice of assay, with studies using ELISA’s for saliva samples and RIA for urinary samples being more likely to base their results on exaggerated cortisol concentrations.

Moreover, inter and intra assay coefficients of variability (CV) were omitted in many studies [40, 44, 48, 49, 53, 56, 60, 65, 70, 75–78, 80, 87, 90, 94]. Typically, it is necessary that samples run on multiple assays, where CV’s refer to the reliability or repeatability of hormone measurements. Thus, failing to report these values raises uncertainty about the cortisol samples obtained. Furthermore, research [98, 99] has consistently shown that inter-assay CVs < 15 % and intra assay CVs of < 10 % are acceptable. However, Salacz et al. (2012) reported an inter-assay coefficient of 16.6 % and higher than accepted inter-assay CV’s might reflect a lack of reliability in the processing of cortisol samples.

### Outcome measures

The use of different depression outcome measures and their limitations may have contributed to the disparity in research findings. Thirty five studies measuring depression utilised self-report questionnaires with only twelve [47, 52, 53, 58, 60, 62, 63, 66, 77, 80, 86, 92] using semi-structured or structured diagnostic interviews. Although using a questionnaire to identify diagnostic status is more time-efficient than an interview, self-report questionnaires are considerably less accurate since skilled clinical interviewers are generally able to probe and check the respondents answers [100]. For instance, high scores on self-report depression scales may be due to other health or mental health concerns than depression. Likewise, self-report measures may confuse high levels in a smaller clusters of symptoms as high levels of depression [100]. Furthermore, studies have used different self-report measures and those utilising the EPDS have adopted different cut-off points for screening depression. For example, Abou-Saleh et al. (1998), O’Keane et al. (2011) and Luiza et al. (2015) used a score of 11 to identify participants experiencing depressive symptoms [79], whereas Nierop et al. (2006) used scores ≥ 9 to recognise probable cases of depression [65]. Similarly, others have used 12 [48, 69, 101], 13 [68] or 10 [73, 93] as the differentiating value and some studies [47, 53, 78] have not reported this cut-off point at all. These differences in cut-off scores can influence the number of depression cases identified and interpretation of overall findings.

Moreover, only 8 studies [47, 53, 58, 60, 62, 63, 66, 92] out of the 11 using semi-structured/structured interviews screened for major depression (as defined by the DSM), with all others basing their results on reports of maternity blues or depressive symptoms. Perinatal blues is a mild transient lability of mood, often associated with tearfulness and low mood [102], whereas major depression is characterized by greater severity and duration. Due to this difference in symptomology, comparisons between the two conditions are difficult and study results are likely to be influenced by the construct being measured.

### Interpretation of main findings

Given the significant discrepancies between studies, it is difficult to draw strong conclusions from these 47 studies. One approach to the interpretation of these studies is to give greater weight to studies with higher quality participant samples, assessment tools, cortisol samples and greater statistical power. Based on an adaption of the Systematic Assessment of Quality in Observational Research (SAQOR) [103], an assessment tool recently developed for evaluating quality in psychiatry research, studies were assessed using the following criteria: 1) whether samples were representative of the population from which they were drawn; 2) whether the source of participant samples was clearly stated; 3) whether participant sampling methods were clearly described (e.g. consecutive, clinical, community, convenience); 4) whether a power calculation for the study’s sample size was included; 5) in accordance with Adam and Kumari (2009), whether the study met the minimum cortisol sampling protocol (three samples in a day), medium sampling protocol (six samples daily or three samples per day over three days) or high sampling protocol (multiple samples per day across several days) 6) if inter and intra assay CV’s were specified and within an acceptable range 7) whether the time cortisol samples were retrieved was reported 8) whether a diagnostic structured/semi-structured interview or a self-report measure with a clearly stated clinical cut-off was used; 9) whether distorting influences such as antidepressant exposure, other mental health disorders and conditions that effect HPA axis function (i.e. Addison’s disease, adrenal insufficiency, Cushing’s disease, congenital adrenal hyperplasia) were considered; 10) effect sizes. Studies were graded on each criteria and given a total score out of a maximum score of 15. Studies were classified as high quality if they screened positive on the majority of the criteria (i.e. received a minimum score of 8), moderate if they obtained a score of 6-7 (screened positive for 33–47 % of the criteria) and low if they obtained a score lower than 6 (see Additional file 2).

According to this classification system, out of the studies not examining the cortisol awakening response, Ehlert et al. (1990), O’Hara et al. (1991), Evans et al. (2008) and Figueiredo et al. (2009) are considered high quality. O’Hara et al. (1991) and Figueiredo et al. (2009) reported no significant relationships between cortisol and perinatal depression. Likewise, Evans et al. (2008) also found no significant associations between depression alone and cortisol levels before or after exposure to a psychophysiology task during late gestation. In contrast, Ehlert et al. (1990) identified higher cortisol concentrations in women exhibiting depression between one to five days postpartum (however, only on days when depressive symptoms were present).

Based on these findings, the most plausible interpretation of currently available literature is that there is not a significant association between cortisol and antenatal depression. However, cortisol’s role in the postpartum still remains uncertain as Ehlert et al. (1990) reported a positive association between cortisol and momentary maternal mood in the immediate postpartum while O’Hara et al. (1991) and Figueiredo et al. (2009) found no significant correlations between cortisol and postpartum mood. This difference in findings might be due to methodological variations between high quality studies investigating the postpartum period. For example, Ehlert et al. (1990) measured salivary free cortisol while O’Hara et al. (1991) and Figueiredo et al. (2009) obtained 24 h urine samples. Furthermore, time of sample collection, gestational period investigated and outcome measures used differ between studies. Given the inconsistency amongst this research and the few high quality studies altogether, further investigation is needed to establish if there is a relationship between cortisol and postpartum depression.

### Cortisol awakening response and maternal depression

In total, 6 studies investigated the CAR-perinatal depression relationship, with 3 [2, 61, 62] identifying significant associations and 3 [67–69] reporting non-significance. Out of these studies, only one study [61] is considered high quality and reported a positive relationship between momentary mood and concurrent cortisol levels (part of the CAR) between 6–37 weeks gestation. However, it should be noted that all 3 studies indicating non-significant associations recruited healthy low-risk participants with no psychiatric disorders and measured self-reported depressive symptoms rather than major depression. In contrast, out of the research indicating significant associations, Taylor et al. (2009) and O’Connor et al. (2014) [2, 62] focused on depressed, high-risk participants and both studies found lower cortisol levels upon waking in women with a diagnosis of depression. Based on these studies, it is suggested that major depressive disorders (rather than maternal blues) may be associated with a blunted cortisol response upon waking in the perinatal period. In support, O’Connor et al. (2014) measured self-reported depressive symptoms and diagnosis of major depression in participants but reported a significant negative relationship only between cortisol and maternal major depression. Thus, studies focusing on the CAR suggest a negative association between major depression and cortisol and a positive association between momentary mood and concurrent cortisol levels.

In sum, the cortisol awakening response is positively related to antenatal momentary mood states and negatively related to major depression in the perinatal period. High quality studies indicate a significant positive relationship between salivary cortisol and depressive symptoms 1–5 days postpartum but non-significant relationships between cortisol and antenatal depression. Major limitations within low quality studies include; limited sampling occasions and investigation of major depressive disorders, insufficient statistical power (due to low number of overall participants and participants with depressive symptoms), differences in substrates and method of cortisol determination between studies.

## Discussion

### Hypercortisolemia

The results do not indicate a positive association between hypercortisolemia and major depression during the antenatal period, with higher quality studies revealing non-significant associations between antenatal depression and cortisol. However, out of the studies investigating CAR, Giesbrecht et al. (2012) indicated a positive relationship between CAR and antenatal momentary mood states. Furthermore, another high quality study [71] found cortisol secretion to be higher among women with postpartum blues than controls. From these findings, a role for hypercortisolemia in transient and momentary negative mood states (but not major depression) seems likely.

### Hypocortisolemia

Out of the eight studies investigating cortisol and postpartum depression, half reported significant negative relationships, [2, 47, 53, 54], while the other half [67, 81, 82, 104] identified non-significant associations. Specifically, significantly lower cortisol levels have been found in depressed women at 3 days, 1 year [53] and 4–6 weeks postpartum [54]. Taylor et al. (2009), the fourth study showing significant results, identified a blunted CAR at 7 weeks postpartum in women with depressive symptoms. Furthermore, Shimazu et al. (2015), reported a negative relationship (although non-significant) between 1 and 3 months postpartum and cortisol. Taken together, these studies indicate an association between hypocortisolemia and depressive symptoms during the postpartum period.

In addition, hypocortisolemia is typically associated with chronic depressive states, and since the majority of studies indicated hypocortisolemia past one month postpartum, the results of this paper support the suggestion that hypocortisolemia is associated with chronic depression. The only study suggesting a significant negative relationship between immediate postpartum depressive symptoms and cortisol produced a negligible effect size (*R*^2^ = -0.0004). Thus, based on this review, chronic depression (past 1 month postpartum) appears to be associated with hypocortisolemia.

However, the majority of studies mentioned above have significant methodological differences and limitations, with only two studies [2, 82] being considered high quality with minimal flaws. In addition, two of the four non-significant studies did not identify measurements of cortisol concentrations for depressed and non-depressed women, reporting only whether associations were significant (*p* < 0.05). Therefore, meaningful findings from these studies such as the direction and relative effect of the relationship may have been overlooked.

Furthermore, hypocortisolemia has often been linked to a particular subtype of depression. Specifically, studies suggest a relationship between hypocortisolemia and atypical depression [105–107], which is defined as involving retention of mood reactivity, weight gain, interpersonal rejection sensitivity, hypersomnia and depressive symptoms that become worse as the day progresses [107]. In contrast, melancholic depression has been associated with hypercortisolemia and is characterised by depressed mood (worse in the morning), reduced appetite and/or substantial weight loss, insomnia and psychomotor alterations [107]. Most studies have not differentiated participants with atypical and melancholic depression. This provides another source of ambiguity and prevents a thorough appraisal of the hypocortisolemia hypothesis.

### Future directions

Future research should aim to include sufficient participants in their designs, report coefficients of variability for cortisol and adhere to the minimum protocols for cortisol sampling occasions to ensure sampling quality and methodological transparency. Specifically, a minimum of three cortisol samples across the day should be obtained to account for cortisol’s diurnal variations. Ideally however, studies should obtain multiple cortisol samples daily and explore patterns of cortisol trajectories (instead of concentrations at single time points). This will help account for individual differences and enhance identification of differences between women with and without depression. There are also limited studies that have measured depression using a diagnostic tool, with most identifying depressive symptoms or maternity blues. Therefore, further investigation into the association between diagnosed major or minor depression in the perinatal period (as identified by a structured or semi-structured interview) and cortisol is required. In addition, a greater number of studies investigating chronic postpartum depression (i.e. up to 12 months) and differences in cortisol levels associated with atypical versus melancholic depression will help in clarifying cortisol’s role in perinatal depression. Lastly, there is limited published data that shows whether depression during pregnancy differs in symptom profile from depression postpartum and whether either differ from depression at other times.

## Conclusions

Given the limitations identified within the research and variations in methodology between studies, generalisations and comparisons are difficult to make. However, the current research indicates that: (1) hypercortisolemia may be associated with immediate postpartum maternal blues or antenatal momentary mood states; and (2) hypocortisolemia is likely to be associated with chronic maternal depressive states extending beyond one month postpartum. Although, as already indicated, higher quality research is required to confirm this association.

These findings are consistent with current literature that suggests hypercortisolemia is positively linked to transient mood lability [22]. In relation to the hypocortisolemia finding, there are two possible explanations 1) withdrawal of the placenta in the postpartum results in hypocortisolemia in women susceptible to chronic major depression [28] 2) initial hypercortisolemia transforms over time into hypocortisolemia during chronic stress, to protect the brain and metabolic processes from prolonged exposure to excess cortisol levels [108]. In support of the second hypothesis, Miller et al. (2007) reviewed studies investigating cortisol levels in a non-pregnant population experiencing chronic stress and revealed that cortisol levels increase at the onset of stressors but subsequently lead to hypocortisolemia as time passes. Miller et al. (2007) further suggested that HPA functioning is influenced by a person’s response to stress, where cortisol levels increase with the extent of subjective distress and are reduced in those who develop PTSD or experience significant trauma. This further highlights that HPA axis function is dependent on length of exposure to stress and nature of the stressor.

## Abbreviations

BDI, becks depression inventory; CAR, cortisol awakening response; CES-D, Centre for Epidemiological Studies Depression Scale; CRH, corticotrophin releasing hormone; CSI, childcare stress inventory; DACL, depressive adjective check list; DISC, diagnostic interview schedule for children; DSM, diagnostic and statistical manual of mental disorders; EDS, Edinburgh depression scale; EIA, enzyme immunoassay; EPDS, Edinburgh postnatal depression scale; GR, glucocorticoid receptors; HAMD, Hamilton rating scale for depression; HPA, hypothalamic-pituitary-adrenal; HRSD, Hamilton rating scale for depression; LES, life experiences survey; MAACL, multiple affect adjective checklist; MADRAS, Montgomery-Asberg depression rating scale; MR, mineralocorticoid receptors; PES, pregnancy experiences scale; POMS, profile of mood states; PSE, present state examination; RIA, radioimmunoassay; SADS, schedule for affective disorders and schizophrenia; SCID, structured clinical inventory for DSM disorders; SCL, symptom checklist; SD, standard deviation; SRDS, Zung self-rating depression scale; TSST, Trier social stress test; VAS, visual analogue scale for mood and anxiety; VMAS, visual analogue mood scales.

## Additional files

Additional file 1: Scopus Search Syntax. This file contains the exact search words used to find relevant articles in the Scopus database for the current systematic literature review. (DOCX 11 kb) Additional file 2: Assessment of the quality of studies using an adaption of the SAQOR criteria. This file contains a table assessing the quality of studies using an adapted version of the Systematic Assessment of Quality in Observational Research. (DOCX 37 kb)
