# Supplementary material for: Perinatal maternal depression and cortisol function in pregnancy and the postpartum period: a systematic literature review
Source: BMC Pregnancy Childbirth. 2016 May 31;16:124. doi: 10.1186/s12884-016-0915-y (PMC4886446; doi:10.1186/s12884-016-0915-y)
Supplement: Additional file 1: — Scopus Search Syntax. This file contains the exact search words used to find relevant articles in the Scopus database for the current systematic literature review. (DOCX 11 kb) [file 12884_2016_915_MOESM1_ESM.docx]

**Additional file 1**

**Syntax from Scopus Search:**

( TITLE-ABS-KEY ( "cortisol concentrations"  OR  cortisol  OR  "cortisol levels"  OR  "steroid hormones" )  AND  TITLE-ABS-KEY ( "perinatal depress*"  OR  "postpartum depress*"  OR  "antenatal depress*"  OR  "baby blues"  OR  "maternal blues"  OR  "maternal depression"  OR  "depress* in pregnancy"  OR  "postnatal depress*" ) )
