# Supplementary material for: Perinatal maternal depression and cortisol function in pregnancy and the postpartum period: a systematic literature review
Source: BMC Pregnancy Childbirth. 2016 May 31;16:124. doi: 10.1186/s12884-016-0915-y (PMC4886446; doi:10.1186/s12884-016-0915-y)
Supplement: Additional file 2: — Assessment of the quality of studies using an adaption of the SAQOR criteria. This file contains a table assessing the quality of studies using an adapted version of the Systematic Assessment of Quality in Observational Research. (DOCX 37 kb) [file 12884_2016_915_MOESM2_ESM.docx]

| **Additional file 2**  *Assessment of the quality of studies using an adaption of the SAQOR criteria* | **Sample representative of population**  Y=1  N=0 | **Sample source**  Y=1  N=0 | **Sampling method**  Y=1  N=0 | **Sample size**  Y=1  N=0 | **Cortisol samples**  1= Minimum standards met  2=Medium standards met  3=High standards met | **Intra and inter assay coefficient of variability (CV).**  0= Not in acceptable range/not identified  1= Both CVs in acceptable range. | **Time cortisol samples were retrieved stated**  Y=1  N=0 | **Measurement of depression/depressive symptoms**  Y=1  N=0 | **Excludes/considers in analysis women with other mental health disorders, use of antidepressants and conditions known to affect HPA axis function.**  1=Considers 1 factor.  2= Considers 2 factors  3=Considers all 3 factors  0= Considers none of these factors. | **Effect size**  0= Small or not stated/insufficient data to obtain effect size.  1=Medium  2=Large | **Quality (total score out of a maximum score of 15)** |
| --- | --- | --- | --- | --- | --- | --- | --- | --- | --- | --- | --- |
| 1. Handley et al. (1977) | Y | Y | N | N | 0 | 1 | N | Y | 0 | 0 | Low (4) |
| 2. Handley et al. (1980). | Y | Y | N | N | 0 | 0 | N | Y | 0 | 0 | Low (3) |
| 3.Babli et al. (1980) | Y | Y | Y | N | 1 | 1 | N | N | 0 | 0 | Low (5) |
| 4. Kuevi et al. (1983). | Y | Y | N | N | 0 | 0 | N | N | 0 | 0 | Low (2) |
| 5.Brinsmead et al. (1985) | Y | N | N | N | 0 | 0 | N | N | 0 | 0 | Low (1) |
| 6. Feksi et al. (1984). | Y | N | N | N | 3 | 0 | Y | Y | 0 | 0 | Moderate (6) |
| 7. Gard et al. (1986) | Y | N | N | N | 0 | 0 | N | Y | 0 | 0 | Low (2) |
| 8. Harris et al. (1989) | Y | Y | N | Y | 0 | 1 | Y | Y | 0 | 0 | Moderate(6) |
| 9. Ehlert et al. (1990). | Y | Y | Y | N | 2 | 1 | Y | Y | 0 | 0 | High (8) |
| 10. Smith et al. (1990) | Y | Y | N | N | 0 | 1 | Y | Y | 0 | 0 | Low (5) |
| 11. O’Hara et al. (1991) | Y | Y | N | N | 3 | 1 | Y | Y | 0 | 0 | High (8) |
| 12. Okano et al. (1992). | Y | Y | N | N | 0 | 1 | Y | Y | 1 | 0 | Moderate (6) |
| 13. Pedersen et al. (1993). | Y | Y | N | N | 0 | 1 | N | Y | 1 | 0 | Low (5) |
| 14. Taylor et al. (1994) | Y | Y | N | N | 0 | 1 | Y | N | 0 | 0 | Low (4) |
| 15. Harris et al. (1994). | Y | Y | N | Y | 2 | 0 | Y | Y | 0 | 0 | Moderate (7) |
| 16. Mahomed et al. (1995) | N | Y | N | N | 0 | 0 | N | N | 0 | 0 | Low (1) |
| 17.Harris et al. (1996) | Y | Y | N | Y | 2 | 0 | Y | Y | 0 | 0 | Moderate (7) |
| 18. Abou-Saleh et al. (1998). | Y | Y | Y | N | 0 | 1 | Y | N | 0 | 0 | Low (5) |
| 19.Lundy et al. (1999) | Y | Y | N | N | 0 | 0 | N | N | 0 | 0 | Low (2) |
| 20. Susman et al. (1999). | Y | Y | N | N | 0 | 1 | Y | N | 0 | 0 | Low (4) |
| 21.Parry et al. (2003) | Y | N | N | N | 2 | 0 | Y | Y | 0 | 0 | Low (5) |
| 22. Field et al. (2004) | Y | Y | N | N | 0 | 1 | N | N | 1 | 0 | Low (4) |
| 23. Diego et al. (2004). | Y | Y | N | N | 0 | 0 | Y | Y | 0 | 2 | Moderate (6) |
| 24.Field et al. (2006) | Y | Y | Y | N | 0 | 1 | N | Y | 1 | 0 | Moderate (6) |
| 25. Nierop et al. (2006). | Y | Y | N | N | 2 | 0 | N | Y | 2 | 0 | Moderate (7) |
| 26. Groer  et al. (2007) | Y | Y | N | N | 0 | 1 | Y | N | 0 | 0 | Low (4) |
| 27. Davis et al. (2007) | Y | Y | N | N | 0 | 1 | Y | N | 0 | 0 | Low (4) |
| 28. Evans et al. (2008). | Y | Y | Y | N | 1 | 1 | Y | Y | 2 | 0 | High (9) |
| 29. Field et al. (2008) | Y | Y | N | N | 0 | 1 | N | Y | 1 | 0 | Low (5) |
| 30.Fan et al. (2009). | Y | Y | Y | N | 0 | 0 | Y | N | 2 | 0 | Moderate (6) |
| 31. Figueiredo et al. (2009). | Y | Y | Y | N | 3 | 1 | N | Y | 0 | 0 | High (8) |
| 32. Yim et al. (2009). | Y | Y | Y | N | 0 | 1 | N | Y | 1 | 0 | Moderate (6) |
| 33. Diego et al. (2009) | Y | Y | N | N | 0 | 1 | N | Y | 2 | 0 | Moderate (6) |
| 34. Cheng et al. (2010) | Y | Y | N | N | 0 | 1 | N | Y | 0 | 0 | Low (4) |
| 35.Taylor et al. (2009) | Y | Y | N | N | 1 | 1 | N | Y | 1 | 1 | Moderate (7) |
| 36. Pluess et al. (2010). | Y | Y | N | N | 1 | 1 | N | Y | 1 | 0 | Moderate (6) |
| 37. Parcells, D.A. (2010) | Y | Y | N | N | 0 | 0 | Y | Y | 1 | 0 | Low (5) |
| 38.O’Keane et al. (2011). | Y | Y | N | N | 0 | 1 | Y | Y | 2 | 0 | Moderate (7) |
| 39. Giesbrecht et al. (2012) | Y | Y | N | N | 2 | 1 | Y | N | 1 | 1 | High (8) |
| 40. Tsubouchi et al. (2011) | Y | Y | N | N | 0 | 1 | Y | Y | 0 | 0 | Low (5) |
| 41. Salacz et al. (2012) | Y | Y | N | N | 0 | 0 | Y | Y | 0 | 0 | Low (4) |
| 42.Voegtime et al.(2013) | Y | Y | N | N | 0 | 1 | Y | Y | 0 | 0 | Low (5) |
| 43. Peer et al. (2013) | N | Y | Y | N | 1 | 1 | Y | Y | 1 | 0 | Moderate (7) |
| 44. Shelton et al. (2014) | Y | Y | N | N | 0 | 1 | Y | N | 1 | 0 | Low (5) |
| 45. O’Connor et al. (2014) | Y | Y | Y | N | 1 | 1 | N | Y | 1 | 0 | Moderate (7) |
| 46. Luiza et al. (2015) | Y | Y | N | N | 0 | 1 | N | Y | 2 | 0 | Moderate (6) |
| 47. Shimizu et al. (2015) | Y | Y | N | N | 0 | 1 | N | Y | 0 | 0 | Low (4) |

***Note.*** **Guide to Scoring:**

**For Sample:** The ‘source’ criterion was met if the paper described where the sample had been recruited from. The ‘sampling method’ criterion was met if study recruitment was defined (i.e. convenience, consecutive, clinical, community). The ‘sample size’ criterion was met if a power analysis calculation was provided for sample size determination, given a specific study hypothesis.

**Cortisol Sampling:** Minimum cortisol sampling standards = three samples in one day; medium cortisol sampling standards= six samples in a day or three samples per day over three days; high cortisol sampling standards = multiple samples per day across several days [79]. The ‘inter and intra assay coefficient of variability (CV)’ criterion was met if the inter-assay CV’s were less than 15 % and intra-assay CV’s were less than 10% [94, 95]. The ‘time cortisol samples were retrieved’ criterion was met if the time at which cortisol samples were obtained was specified.

**Measurement of Depression:** This criterion was met if a diagnostic tool or a self-report measure with a clear clinical cut off score was used in the measurement of depression or depressive symptoms.

**Quality (total score out of a maximum score of 15):** Studies screening positive for above 50% of the criteria (i.e. a score of 8/15) = high quality; studies screening positive for 33-46% of the criteria (i.e. a score of 5-6/15) = moderate quality; studies screening positive for below 33% of the criteria (i.e. a score of less than 5) = low quality.
